# Supplementary material for: How Jaminan Kesehatan Nasional (JKN) coverage influences out-of-pocket (OOP) payments by vulnerable populations in Indonesia
Source: PLOS Glob Public Health. 2022 Jul 7;2(7):e0000203. doi: 10.1371/journal.pgph.0000203 (PMC10021284; doi:10.1371/journal.pgph.0000203)
Supplement: S1 Table — This table is the extended version of Table 1, which observes 3 household population segments; uninsured, JKN, and private and mixed insurance (households whose members has a variety of insurance, e.g: JKN and private insurance) with both weighted and unweighted totals. These segments are broken down by all variables used in the regression model. (DOCX) [file pgph.0000203.s001.docx]

# **Supporting Information**

**S1 Table.** **Descriptive Statistics by Insurance Types**

| Variable | Uninsured | | JKN Household | | Private & mixed | |
| --- | --- | --- | --- | --- | --- | --- |
|  | UW | W | UW | W | UW | W |
| Total Observation: | 155,071 | 38,354,062 | 318,768 | 69,018,564 | 136,151 | 33,670,790 |
| Explanatory variables: |  |  |  |  |  |  |
| Medical use: |  |  |  |  |  |  |
| Never out/inpatient | 104,805 | 24,932,828 | 176,308 | 37,195,449 | 66,378 | 15,956,705 |
| Outpatient only at public hospital | 801 | 168,160 | 5,511 | 1,129,328 | 1,806 | 404,293 |
| Outpatient only at private hospital | 822 | 261,935 | 3,609 | 1,089,479 | 1,755 | 614,545 |
| Outpatient only at public PHC | 11,079 | 2,499,993 | 38,448 | 6,913,912 | 15,672 | 3,106,322 |
| Outpatient only at private PHC | 24,788 | 7,021,709 | 38,379 | 10,030,850 | 19,194 | 5,581,954 |
| Outpatient only at mixed facilities | 1,011 | 301,913 | 3,424 | 830,818 | 2,333 | 667,178 |
| Inpatient only at public hospital | 1,966 | 397,901 | 11,738 | 2,181,665 | 5,681 | 1,106,884 |
| Inpatient only at private hospital | 1,644 | 509,474 | 6,732 | 1,913,726 | 3,984 | 1,297,578 |
| Inpatient only at public PHC | 1,226 | 295,086 | 3,004 | 584,490 | 2,346 | 478,295 |
| Inpatient only at private PHC | 1,016 | 309,220 | 1,175 | 323,310 | 1,111 | 351,409 |
| Inpatient only at mixed facilities | 51 | 12,540 | 276 | 58,065 | 174 | 46,153 |
| In & outpatient at public hospital | 450 | 103,154 | 4,981 | 1,000,758 | 1,786 | 396,364 |
| In & outpatient at private hospital | 505 | 156,880 | 2,865 | 864,476 | 1,428 | 527,298 |
| In & outpatient at public PHC | 584 | 128,257 | 2,625 | 461,468 | 1,600 | 300,957 |
| In & outpatient at private PHC | 839 | 257,250 | 881 | 249,139 | 745 | 244,148 |
| In & outpatient at mixed facilities | 3,484 | 997,762 | 18,812 | 4,191,631 | 10,158 | 2,590,707 |
| Health status (at least one HH member feeling sick in past one month): | | | | | | |
| No | 67,123 | 15,946,654 | 125,912 | 26,280,499 | 45,785 | 11,103,640 |
| Yes | 87,948 | 22,407,408 | 192,856 | 42,738,065 | 90,366 | 22,567,150 |
| Location: | | | | | | |
| Urban | 55,204 | 18,080,095 | 143,629 | 40,594,146 | 58,139 | 19,489,078 |
| Rural | 99,867 | 20,273,967 | 175,139 | 28,424,418 | 78,012 | 14,181,712 |
| Education of household head: | | | | | | |
| At most Primary school | 90,518 | 21,926,971 | 159,646 | 33,383,022 | 67,741 | 16,171,978 |
| Junior high school | 26,920 | 6,877,670 | 45,967 | 10,235,720 | 20,863 | 5,266,791 |
| Senior high school | 27,421 | 6,683,742 | 66,069 | 14,669,640 | 27,897 | 6,776,916 |
| University | 10,212 | 2,865,679 | 47,086 | 10,730,182 | 19,650 | 5,455,105 |
| Household size | 155,071 | 38,354,062 | 318,768 | 69,018,564 | 136,151 | 33,670,790 |
| Household Quintile: |  |  |  |  |  |  |
| Q1 | 41,730 | 9,673,958 | 71,582 | 14,046,796 | 20,778 | 4,421,884 |
| Q2 | 35,647 | 8,614,071 | 63,266 | 13,308,171 | 27,073 | 6,290,487 |
| Q3 | 31,811 | 7,918,084 | 62,171 | 13,529,769 | 27,931 | 6,778,199 |
| Q4 | 27,336 | 6,985,923 | 62,117 | 13,910,950 | 28,910 | 7,330,799 |
| Q5 | 18,547 | 5,162,026 | 59,632 | 14,222,878 | 31,459 | 8,849,421 |
| Year: |  |  |  |  |  |  |
| 2018 | 78,263 | 19,543,065 | 150,018 | 33,559,008 | 66,491 | 16,763,662 |
| 2019 | 76,808 | 18,810,997 | 168,750 | 35,459,556 | 69,660 | 16,907,128 |
| Household head gender: |  |  |  |  |  |  |
| Male | 131,026 | 32,580,504 | 266,396 | 57,717,659 | 118,133 | 29,201,144 |
| Female | 24,045 | 5,773,558 | 52,372 | 11,300,905 | 18,018 | 4,469,646 |
| Household head age | 155,071 | 38,354,062 | 318,768 | 69,018,564 | 136,151 | 33,670,790 |
| Household head occupation: |  |  |  |  |  |  |
| Not working | 15,017 | 3,997,162 | 41,996 | 10,126,108 | 15,599 | 4,230,064 |
| Farming, fisheries & livestock | 68,166 | 13,667,856 | 115,151 | 18,968,472 | 49,515 | 9,123,078 |
| Minning | 2,693 | 440,747 | 4,599 | 712,530 | 3,233 | 540,607 |
| Manufacture | 9,668 | 3,141,644 | 20,622 | 6,384,226 | 11,075 | 3,932,701 |
| Construction/infrastructure | 12,364 | 3,592,760 | 22,927 | 5,666,687 | 11,431 | 3,201,222 |
| Trade & services | 46,924 | 13,431,718 | 112,927 | 27,004,323 | 45,124 | 12,581,130 |
| Others | 239 | 82,175 | 546 | 156,218 | 174 | 61,988 |
| Housing floor material: |  |  |  |  |  |  |
| Traditional (wood, soil) | 86,504 | 16,080,049 | 173,224 | 27,519,579 | 73,938 | 13,002,110 |
| Modern (granite/ceramics/vinyl/tiles) | 68,567 | 22,274,013 | 145,544 | 41,498,985 | 62,213 | 20,668,680 |
| Housing defecation facility: |  |  |  |  |  |  |
| Do not have | 8,461 | 1,619,532 | 15,312 | 2,393,292 | 6,924 | 1,301,742 |
| Private | 118,705 | 30,093,225 | 251,467 | 56,024,460 | 106,316 | 27,094,012 |
| Sharing | 27,905 | 6,641,305 | 51,989 | 10,600,812 | 22,911 | 5,275,036 |
| Region: |  |  |  |  |  |  |
| Sumatera | 47,812 | 8,735,262 | 92,515 | 14,176,194 | 33,578 | 6,255,816 |
| Jawa-Banten | 51,288 | 22,496,556 | 99,034 | 40,614,772 | 42,185 | 19,877,450 |
| Bali-NT | 11,261 | 2,109,156 | 21,532 | 3,131,510 | 13,879 | 2,274,659 |
| Kalimantan | 17,911 | 2,481,603 | 29,756 | 4,101,495 | 12,559 | 1,836,908 |
| Sulawesi | 16,633 | 1,852,954 | 43,970 | 4,914,834 | 22,847 | 2,726,389 |
| Maluku-Papua | 10,166 | 678,531 | 31,961 | 2,079,759 | 11,103 | 699,568 |
