# Supplementary material for: How Jaminan Kesehatan Nasional (JKN) coverage influences out-of-pocket (OOP) payments by vulnerable populations in Indonesia
Source: PLOS Glob Public Health. 2022 Jul 7;2(7):e0000203. doi: 10.1371/journal.pgph.0000203 (PMC10021284; doi:10.1371/journal.pgph.0000203)
Supplement: S2 Table — This table depicts the regression output of the two-part model, where the first part is a logit model with the binary dependent variable of zero and positive OOP payments. It estimates the likelihood of a household incurring zero or positive OOP payments. The second part is a Generalized Linear Model (GLM) with gamma error distribution and a log link function. It estimates the level or intensity of OOP payment, conditional on a household spending anything OOP. (DOCX) [file pgph.0000203.s002.docx]

# **Supporting Information**

**S2 Table. Two Part Model Output**

| **Independent Variables** | **Dependent Variable** | | | |  |
| --- | --- | --- | --- | --- | --- |
|  | **Part I- Probability of Positive OOP Expenditure** | | **Part II-**  **Average OOP Expenditure** | |  |
|  |  |  |  |  |  |
|  | Coeff | Std. Error | Coeff | Std. Error |  |
| Insurance type: |  |  |  |  |  |
| No insurance (ref) |  |  |  |  |  |
| JKN only | 0.00276** | 0.001 | -0.143*** | 0.001 |  |
| Private & Mixed | 0.243*** | 0.001 | 0.122*** | 0.001 |  |
| Household Consumption Quintiles |  |  |  |  |  |
| 1^st^ quintile (ref) |  |  |  |  |  |
| 2nd quintile | 0.269*** | 0.001 | 0.455*** | 0.001 |  |
| 3rd quintile | 0.399*** | 0.001 | 0.779*** | 0.001 |  |
| 4th quintile | 0.440*** | 0.001 | 1.150*** | 0.001 |  |
| 5th quintile | 0.480*** | 0.001 | 1.915*** | 0.002 |  |
| Type of Service & Provider Source |  |  |  |  |  |
| Never in/outpatient (ref) |  |  |  |  |  |
| outpatient only at public hospital | 2.684*** | 0.011 | 1.146*** | 0.003 |  |
| outpatient only at private hospital | 3.449*** | 0.013 | 1.250*** | 0.004 |  |
| outpatient only at public PHC | 1.837*** | 0.002 | -0.0424*** | 0.001 |  |
| outpatient only at private PHC | 2.762*** | 0.002 | 0.303*** | 0.001 |  |
| outpatient only at mixed facilities | 2.976*** | 0.011 | 0.523*** | 0.003 |  |
| inpatient only at public hospital | 3.141*** | 0.008 | 2.360*** | 0.002 |  |
| inpatient only at private hospital | 4.193*** | 0.013 | 2.604*** | 0.002 |  |
| inpatient only at public PHC | 3.609*** | 0.013 | 1.362*** | 0.002 |  |
| inpatient only at private PHC | 2.889*** | 0.010 | 1.759*** | 0.002 |  |
| inpatient only at mixed facilities | 2.121*** | 0.040 | 2.478*** | 0.008 |  |
| In & outpatient at public hospital | 3.408*** | 0.021 | 2.408*** | 0.005 |  |
| In & outpatient at private hospital | 3.858*** | 0.022 | 2.687*** | 0.003 |  |
| In & outpatient at public PHC | 3.737*** | 0.023 | 1.220*** | 0.003 |  |
| In & outpatient at private PHC | 3.264*** | 0.014 | 1.894*** | 0.002 |  |
| In & outpatient at mixed facilities | 4.034*** | 0.010 | 2.338*** | 0.001 |  |
| JKN and Wealth Quintiles |  |  |  |  |  |
| JKN only - 1st quintile (ref) |  |  |  |  |  |
| JKN only - 2nd | -0.159*** | 0.001 | 0.0013 | 0.001 |  |
| JKN only - 3rd | -0.220*** | 0.001 | -0.0115*** | 0.001 |  |
| JKN only - 4th | -0.152*** | 0.001 | 0.0149*** | 0.001 |  |
| JKN only - 5th | -0.0743*** | 0.001 | 0.0424*** | 0.002 |  |
| Insurance & Service Type at Provider Source |  |  |  |  |  |
| No insurance – Never in/outpatient (ref) |  |  |  |  |  |
| JKN only – outpatient only at public hospital | -1.743*** | 0.011 | -0.389*** | 0.004 |  |
| JKN only – outpatient only at private hospital | -2.229*** | 0.013 | -0.276*** | 0.005 |  |
| JKN only – outpatient only at public PHC | -1.263*** | 0.002 | -0.00166 | 0.002 |  |
| JKN only – outpatient only at private PHC | -0.825*** | 0.002 | 0.00544*** | 0.001 |  |
| JKN only – outpatient only at mixed facilities | -1.075*** | 0.012 | -0.102*** | 0.003 |  |
| JKN only – inpatient only at public hospital | -1.832*** | 0.008 | -0.539*** | 0.002 |  |
| JKN only – inpatient only at private hospital | -2.591*** | 0.013 | -0.451*** | 0.002 |  |
| JKN only – inpatient only at public PHC | -2.388*** | 0.014 | -0.335*** | 0.003 |  |
| JKN only – inpatient only at private PHC | -0.772*** | 0.012 | -0.0134*** | 0.004 |  |
| JKN only – inpatient only at mixed facilities | -0.161*** | 0.042 | -0.121*** | 0.010 |  |
| JKN only – in & outpatient at public hospital | -2.349*** | 0.021 | -0.382*** | 0.006 |  |
| JKN only – in & outpatient at private hospital | -2.483*** | 0.022 | -0.471*** | 0.003 |  |
| JKN only – in & outpatient at public PHC | -2.773*** | 0.024 | -0.250*** | 0.004 |  |
| JKN only – in & outpatient at private PHC | -0.949*** | 0.017 | -0.168*** | 0.004 |  |
| JKN only – in & outpatient at mixed facilities | -2.461*** | 0.010 | -0.461*** | 0.002 |  |
| Insurance & Urban vs Rural |  |  |  |  |  |
| No insurance – Rural (ref) |  |  |  |  |  |
| JKN only – Urban | 0.0395*** | .001 | 0.0563*** | 0.001 |  |
| Private & Mixed – Urban | 0.0149*** | .001 | 0.0647*** | 0.001 |  |
| Province |  |  |  |  |  |
| Aceh (ref) |  |  |  |  |  |
| North Sumatera | -0.0261*** | 0.0018 | 0.240*** | 0.002 |  |
| West Sumatera | -0.225*** | 0.002 | 0.0517*** | 0.003 |  |
| Riau | 0.104*** | 0.002 | 0.186*** | 0.002 |  |
| Jambi | 0.285*** | 0.002 | 0.153*** | 0.002 |  |
| South Sumatera | 0.539*** | 0.002 | 0.159*** | 0.002 |  |
| Bengkulu | 0.330*** | 0.002 | 0.108*** | 0.003 |  |
| Lampung | 0.578*** | 0.002 | 0.220*** | 0.001 |  |
| Bangka Belitung | 0.436*** | 0.003 | 0.0540*** | 0.003 |  |
| Riau Islands | -0.267*** | 0.002 | 0.183*** | 0.003 |  |
| Jakarta | 0.0901*** | 0.001 | 0.106*** | 0.002 |  |
| West Java | 0.485*** | 0.001 | 0.166*** | 0.001 |  |
| Central Java | 0.239*** | 0.001 | 0.219*** | 0.001 |  |
| Yogyakarta | -0.103*** | 0.002 | 0.0222*** | 0.002 |  |
| East Java | 0.403*** | 0.001 | 0.362*** | 0.001 |  |
| Banten | 0.493*** | 0.002 | 0.0609*** | 0.001 |  |
| Bali | 0.0242*** | 0.002 | 0.184*** | 0.002 |  |
| West Nusa Tenggara | 0.602*** | 0.002 | -0.0796*** | 0.002 |  |
| East Nusa Tenggara | -1.073*** | 0.002 | -0.244*** | 0.002 |  |
| West Kalimantan | 0.421*** | 0.002 | 0.228*** | 0.002 |  |
| Central Kalimantan | 0.259*** | 0.002 | 0.179*** | 0.002 |  |
| South Kalimantan | 0.346*** | 0.002 | 0.158*** | 0.002 |  |
| East Kalimantan | -0.0589*** | 0.002 | 0.0867*** | 0.002 |  |
| North Kalimantan | -0.341*** | 0.004 | 0.0453*** | 0.004 |  |
| North Sulawesi | -0.181*** | 0.002 | 0.107*** | 0.003 |  |
| Central Sulawesi | -0.384*** | 0.002 | -0.187*** | 0.002 |  |
| South Sulawesi | -0.661*** | 0.002 | -0.0843*** | 0.002 |  |
| Southeast Sulawesi | -0.580*** | 0.002 | -0.155*** | 0.003 |  |
| Gorontalo | -0.237*** | 0.003 | -0.346*** | 0.002 |  |
| West Sulawesi | -1.134*** | 0.003 | -0.238*** | 0.004 |  |
| Maluku | -0.888*** | 0.003 | -0.0864*** | 0.003 |  |
| North Maluku | -0.825*** | 0.003 | -0.00690 | 0.004 |  |
| West Papua | -1.095*** | 0.003 | -0.169*** | 0.005 |  |
| Papua | -0.919*** | 0.002 | -0.227*** | 0.002 |  |
| Education level |  |  |  |  |  |
| Primary school (ref) |  |  |  |  |  |
| Junior high school | -0.0310*** | 0.000 | 0.0183*** | 0.000 |  |
| Senior high school | -0.181*** | 0.000 | 0.0203*** | 0.000 |  |
| University | -0.249*** | 0.000 | 0.111*** | 0.000 |  |
| Household Size | 0.189*** | 0.000 | -0.100*** | 0.000 |  |
| Robust standard errors | | | | |  |
| *** p<0.01, ** p<0.05, * p<0.1 | | | | |  |

Note: We use *margins* command in Stata 16 to obtain average marginal effects
