# Supplementary material for: How Jaminan Kesehatan Nasional (JKN) coverage influences out-of-pocket (OOP) payments by vulnerable populations in Indonesia
Source: PLOS Glob Public Health. 2022 Jul 7;2(7):e0000203. doi: 10.1371/journal.pgph.0000203 (PMC10021284; doi:10.1371/journal.pgph.0000203)
Supplement: S3 Table — This table shows the marginal results of the regression output; the first part, and the second part which consists of conditional and unconditional effects. The marginal result of the first part shows households’ probability of paying OOP, the conditional effect shows the average OOP spending conditional on households having spent on OOP, and the unconditional effect shows the estimated effect on the entire population. (DOCX) [file pgph.0000203.s003.docx]

# **Supporting Information**

**S3 Table. Marginal Results by Key Variables**

| **Variable** | **Part I- Probability of Positive OOP Expenditure^a^ (%)** | **Conditional Part II-**  **Average OOP Expenditure (IDR)^b^** | **Unconditional Part II-**  **Average OOP Expenditure (IDR)^c^** |
| --- | --- | --- | --- |
| **Insurance type:** | | | |
| No insurance | 0.733 | 1,420,242 | 1,041,037 |
| JKN only | 0.663 | 960,350 | 636,712 |
| Private & Mixed | 0.704 | 1,042,686 | 734,050 |
| **Insurance & Wealth Quintiles:** | | | |
| No insurance - 1st | 0.689 | 416,179 | 286,747 |
| No insurance - 2nd | 0.729 | 656,216 | 478,382 |
| No insurance - 3rd | 0.748 | 907,630 | 678,907 |
| No insurance - 4th | 0.753 | 1,314,213 | 989,602 |
| No insurance - 5th | 0.759 | 2,824,893 | 2,144,094 |
| JKN only - 1st | 0.628 | 280,845 | 176,371 |
| JKN only - 2nd | 0.648 | 443,403 | 287,325 |
| JKN only - 3rd | 0.661 | 605,503 | 400,237 |
| JKN only - 4th | 0.681 | 900,268 | 613,083 |
| JKN only - 5th | 0.701 | 1,988,770 | 1,394,128 |
| Private & Mixed - 1st | 0.675 | 332,350 | 224,336 |
| Private & Mixed - 2nd | 0.706 | 466,154 | 329,105 |
| Private & Mixed - 3rd | 0.707 | 663,626 | 469,184 |
| Private & Mixed - 4th | 0.718 | 952,840 | 684,139 |
| Private & Mixed - 5th | 0.716 | 2,194,570 | 1,571,312 |
| **Insurance & Service Type at Provider Source:** | | | |
| No insurance – outpatient only at public hospital | 0.945 | 1,300,694 | 1,229,156 |
| No insurance – outpatient only at private hospital | 0.973 | 1,443,739 | 1,404,758 |
| No insurance – outpatient only at public PHC | 0.884 | 396,217 | 350,256 |
| No insurance – outpatient only at private PHC | 0.949 | 559,780 | 531,231 |
| No insurance – outpatient only at mixed facilities | 0.958 | 697,847 | 668,538 |
| No insurance – inpatient only at public hospital | 0.964 | 4,379,311 | 4,221,656 |
| No insurance – inpatient only at private hospital | 0.987 | 5,589,804 | 5,517,137 |
| No insurance – inpatient only at public PHC | 0.977 | 1,615,136 | 1,577,988 |
| No insurance – inpatient only at private PHC | 0.954 | 2,402,338 | 2,291,830 |
| No insurance – inpatient only at mixed facilities | 0.909 | 4,928,467 | 4,479,977 |
| No insurance – in & outpatient at public hospital | 0.972 | 4,593,286 | 4,464,674 |
| No insurance – in & outpatient at private hospital | 0.982 | 6,074,057 | 5,964,724 |
| No insurance – in & outpatient at public PHC | 0.98 | 1,400,253 | 1,372,248 |
| No insurance – in & outpatient at private PHC | 0.968 | 2,747,352 | 2,659,437 |
| No insurance – in & outpatient at mixed facilities | 0.985 | 4,285,866 | 4,221,578 |
| JKN only – outpatient only at public hospital | 0.745 | 809,829 | 603,322 |
| JKN only – outpatient only at private hospital | 0.791 | 1,005,989 | 795,737 |
| JKN only – outpatient only at public PHC | 0.677 | 363,296 | 245,951 |
| JKN only – outpatient only at private PHC | 0.881 | 516,893 | 455,382 |
| JKN only – outpatient only at mixed facilities | 0.878 | 578,915 | 508,287 |
| JKN only – inpatient only at public hospital | 0.805 | 2,345,490 | 1,888,119 |
| JKN only – inpatient only at private hospital | 0.844 | 3,270,201 | 2,760,050 |
| JKN only – inpatient only at public PHC | 0.791 | 1,061,478 | 839,629 |
| JKN only – inpatient only at private PHC | 0.898 | 2,176,772 | 1,954,741 |
| JKN only – inpatient only at mixed facilities | 0.884 | 4,010,663 | 3,545,426 |
| JKN only – in & outpatient at public hospital | 0.766 | 2,879,454 | 2,205,662 |
| JKN only – in & outpatient at private hospital | 0.814 | 3,484,011 | 2,835,985 |
| JKN only – in & outpatient at public PHC | 0.75 | 1,000,979 | 750,734 |
| JKN only – in & outpatient at private PHC | 0.914 | 2,133,680 | 1,950,184 |
| JKN only – in & outpatient at mixed facilities | 0.841 | 2,482,358 | 2,087,663 |
| Private & Mixed – outpatient only at public hospital | 0.818 | 854,524 | 699,001 |
| Private & Mixed – outpatient only at private hospital | 0.804 | 929,901 | 747,640 |
| Private & Mixed – outpatient only at public PHC | 0.756 | 431,046 | 325,871 |
| Private & Mixed – outpatient only at private PHC | 0.898 | 561,431 | 504,165 |
| Private & Mixed – outpatient only at mixed facilities | 0.913 | 640,914 | 585,154 |
| Private & Mixed – inpatient only at public hospital | 0.836 | 2,442,886 | 2,042,253 |
| Private & Mixed – inpatient only at private hospital | 0.872 | 3,250,366 | 2,834,319 |
| Private & Mixed – inpatient only at public PHC | 0.827 | 1,076,035 | 889,881 |
| Private & Mixed – inpatient only at private PHC | 0.916 | 2,079,119 | 1,904,473 |
| Private & Mixed – inpatient only at mixed facilities | 0.842 | 5,008,291 | 4,216,981 |
| Private & Mixed – in & outpatient at public hospital | 0.798 | 3,555,861 | 2,837,577 |
| Private & Mixed – in & outpatient at private hospital | 0.783 | 3,976,512 | 3,113,609 |
| Private & Mixed – in & outpatient at public PHC | 0.815 | 1,000,694 | 815,566 |
| Private & Mixed – in & outpatient at private PHC | 0.94 | 1,909,435 | 1,794,869 |
| Private & Mixed – in & outpatient at mixed facilities | 0.897 | 2,564,878 | 2,300,696 |
| **Insurance & Rural:** | | | |
| No insurance – Rural | 0.768 | 1,544,360 | 1,186,068 |
| JKN only – Rural | 0.702 | 1,007,569 | 707,313 |
| Private & Mixed – Rural | 0.743 | 1,088,174 | 808,513 |
| **Insurance & Rural:** |  |  |  |
| No insurance – Urban | 0.705 | 1,352,679 | 953,639 |
| JKN only – Urban | 0.63 | 933,667 | 588,210 |
| Private & Mixed – Urban | 0.671 | 1,016,834 | 682,296 |

Notes:

a = the results show the probability of paying OOP, while the probability of not paying OOP is obtained from subtracting 1 with the results in the table. For example, the probability of JKN only households paying OOP is 0.663, while their probability of not paying OOP is 0.34.

b = the results show the average OOP spending conditional on households having spent on OOP. For example. JKN only household have less average OOP spending (IDR 960,350) than uninsured household (IDR 1,420,242) by 32%.

c = In order to get an estimate at the population level, the marginal effect from the first part (a) is multiplied with the marginal effect from the second part (b). For example, JKN only household in the third wealth quintile (IDR 605,503) have less average OOP spending than uninsured household (IDR 907,630) in the same quintile by 41%.
