# Supplementary material for: How Jaminan Kesehatan Nasional (JKN) coverage influences out-of-pocket (OOP) payments by vulnerable populations in Indonesia
Source: PLOS Glob Public Health. 2022 Jul 7;2(7):e0000203. doi: 10.1371/journal.pgph.0000203 (PMC10021284; doi:10.1371/journal.pgph.0000203)
Supplement: Attachment — Submitted filename: Response to Reviewers.docx [file pgph.0000203.s004.docx]

Thank you to the reviewers for their thorough read and astute comments and requests for clarification. We feel the comments and questions have helped us to clarify and, in some cases, simplify our descriptions. Please find our responses in blue to the feedback below.

**Feedback from Reviewer 1:**

*Comments to the author:*

The paper needs some significant modification, for it to be published. There are problems in choosing the key outcome variable(s) and suggested change might potentially change the entire results. However, the study has its significant and needs to be published with modifications.

Specific comments:

1. Please mention the unweighted sample size (pp4, line112)

Thank you for this feedback. We have added the unweighted sample size in line 127 – 129. Without sample weight, the 2018 data consists of 1,131,825 individuals and 295,155 households, while the 2019 data consists of 1,204,466 individuals and 315,672 households.

2. Exclusion of non-prescription medicines and traditional medicine from overall assessment of OOP provides an incomplete picture of OOP. Literature suggest that it is the poorer sections of the society and those living in rural areas would be using these types of care more than the richer section living in urban areas, working in the formal sector. Thus in figure 1 where share of OOP as % of household non-food expenditure is being captured, these expenses need to be included to provide a comprehensive expenditure and results are biased against the poorer sections, showing lower hardship on their part. The author could provide a comparative picture of OOP including and excluding these elements, while limiting to OOP incurred in modern facilities for regression results.

Thank you for this comment. We were not clear in the original manuscript that Figure 1 covers all components of OOP including traditional medicine, and non-prescribed medicines. They are only excluded in the regression part because our goal is to estimate the influence of JKN on health services that are indeed included in the JKN benefit package. Line 263- 264 have incorporated the description. We have also incorporated your suggestion to present the figure with and without these expenses, thus Figure 1 is replaced with:

3. It is important to know the extent of free care accessed by households, as this is a key goal of the JKN program. However, treating all other expenses as same (considering them as positive as part of the binary outcome variable) is a major limitation of the study, as this fails to capture the effect of the scheme in reducing financial hardship.

Thank you for this feedback and we understand your point. Here, we are trying to convey that all other expenses (traditional & non-prescribed meds) are not accounted for in the regression model because they are not included in the JKN benefit package. Our goal is to estimate the influence of JKN on health services that are indeed included in the JKN benefit package. Therefore, including other expenses in the regression would cause a misleading result. We agree that the key goal of JKN is to provide free care access by households, but free healthcare is only covered through health facilities based on medical indications. In the context of reducing financial hardship as a whole, a different study is needed to see if JKN is able to encourage people to abandon visits to traditional practices and avoid buying non-prescribed meds.

4. Similarly, absolute OOP also does not make much sense unless adjusted for households' ability to pay.

Thank you for your comment. We have tried to adjust OOP to real terms with the aim of reflecting ability to pay and comparability across regions. OOP health spending was adjusted in real terms using the ratio of the national average poverty line in a base year (2018) over the district’s poverty line for the given year.

5.Financial hardship can be better assessed if the authors had taken catastrophic incidences (at various thresholds) as these are adjusted for households' ability to pay.

Thank you for your comment. As Susenas 2018 is the first study in Indonesia to provide OOP data, our goal was to first assess whether JKN is on the right track since its implementation in 2014 by estimating its influence on health services that are in the benefit package based on wealth quintiles, health providers, and regions. We believe that this finding would encourage improvements in the JKN system to achieve UHC and gives financial protection. Looking at catastrophic incidence is very important and will be a potential topic for our next study. But we think it goes beyond the more narrower scope of this study.

6. A stated limitation of the SUSENAS data is it doesn't provide any information on the intensity of use of hospitalization, times used, days of stay etc. It needs to be noted that there exists possibilities that the same family might use different types of facilities for different incidence of hospitalization and hence have different outcomes of OOP. this would significantly affect the results. This needs to be specified.

We have calculated and incorporated your input, in the new Figure 4 in line 331. Each household is classified depending on where members of the household come for outpatient and inpatient care. If household members visit several types of providers in that year, then they are categorized as 'mixed'.

7. The characteristics of households who are covered under the JKN and who are not could be very different, thus creating selection biases. It is important that the authors provide a summary table, stating the socio-economic characteristics of households based on coverage under various insurance schemes and compare those who are not covered.

Thank you for your feedback. We agree that there might be a potential bias/ endogeneity due to people self-select their insurance. However, as we explained in line 226, it is argued that since the JKN enrolment is mandatory, this would not cause a major concern. We refer to existing studies:

^33^ Waters, H. R. (1999). "Measuring the impact of health insurance with a correction for selection bias--a case study of Ecuador." Health Econ 8(5): 473-83.

^34^ Zhang, S., Wang, D., Yu, S.: Precise large deviations of aggregate claims in a size-dependent renewal risk model with stopping time claim-number process. J. Inequal. Appl. 2017, 82 (2017))

For the summary table, we propose table 1 is sufficient to give an overview (line 255). A table comparing groups of households would be too long, and would not address the issue since JKN enrolment is mandatory. We have included this in the supporting information section (line 617).

**Feedback from Reviewer 2:**

*Comments to the author:*
The above manuscript makes new attempt at capturing the impact of JKN (national health insurance) on households’ OOP spending in Indonesia. This was made possible by an improved household tool capturing OOP payment in SUSENAS (National Socio-Economic Survey) beginning from 2018.
Following are the observations that the authors may want to address to improve the quality of the manuscript.

1. Scheme design: JKN scheme design is unclear from the reading of the manuscript. For instance, are there exclusion or inclusion criteria in the scheme (number of household members included or excluded from the scheme) and the scheme benefit is unknown:)

i) What benefits are provided (benefit package that contains only inpatient and outpatient or only inpatient)?

ii) how many clinical/medical interventions are included in the scheme?;

iii) are there caps imposed per households per annum on the benefits?

iv) Are medications provided free?

v) And what are additional benefits added during the implementation of JKN as against pre-JKN era, especially in the public facilities?

vi) If benefits are comprehensive, do these facilities use negative list (benefits not covered) or positive list (number of packages covered by the scheme).

Thank you for this feedback. We have added these details for these points in lines 91-102.

2. The methods section outlines sample size indicating 263,666,217 individuals (69,954,912 households). Although it correctly identifies this number to be sample weighted numbers, the key sample size that must be reported is about sample size without weights. Otherwise, it gives an impression that as if the entire Indonesian population is sampled in the survey, which is nothing short of a census, and that is not the case here. And are these samples collected in one period (March) is the total sample or half of the annual sample?

Thank you for your feedback and it is a good point. We have incorporated the unweighted sample in Table 1(line 255). Regarding your comment on the March and September sample size, we have elaborated on this point in the first paragraph of the method section (line 127-129). The sampling design for March is conducted to fully represent district/ city level, while the September one aims to update socio-economic conditions, so that it uses a different set of samples which could be representative until province level. Both datasets are cross-sectional and cannot be merged.

3. The methods section did not clearly spell out whether the survey captured information about inpatient and outpatient expenditure separately. If so, what was the recall period for Inpatient and Outpatient? Did the survey also collect information about non-medical expenditure including lodging, transport, etc. because these expenditure can be considerably higher among those in inaccessible and underprivileged areas, an objective which JKN seeks to bridge inequity.

Thank you for this feedback. Inpatient and outpatient expenditure is recalled annually. Information about non-medical expenditure OOP is only available for transport cost to facilities. However, we exclude this because we only focus on the effect of JKN’s benefits (i.e. medical services) on OOP. We elaborated this in line 137-143.

4. Statistical Analysis: Page 9: If most part of the population are covered by the sample, and assuming such a large sample, it is unlikely that the authors did not obtain adequate samples to disaggregate providers by public and private – due to limited observations. This needs explanation. It is equally important to consider the fact that even after being covered by health insurance, households do end up paying out of pocket, especially in private facilities.

Thank you for bringing up this point. For national level analysis, we did disaggregate providers by public and private. However, in the regional analysis, we could not do this due to limited observations because many observations do not access inpatient or outpatient care in health facilities, especially private PHC in Eastern regions (line 242-243).

5. Page 11 - First Part Model Results: What accounts or reasons for an uninsured (27%) not paying OOP despite being ill. It is unclear whether all of those uninsured utilised public facilities and for free? This requires explanation.

Thank you for this comment. It is interesting that the uninsured have the probability to not pay OOP (27%), which may be due to some districts’ local regulation on providing healthcare support if their residents can prove their eligibility, for example Papua allows its native residents to access free healthcare at most facilities. We have added this explanation in line 276 - 279

6. Page 12: Despite being covered, what explains JKN enrolled population still paying OOP, even though they may be paying less than an uninsured?

Thank you for this feedback. We would like to point you to our findings on this in the discussion section (see lines below). There are some factors that explains this phenomenon:

1. Lack of knowledge and barrier to access (line 383-385)
2. Stronger preference for private facilities or types of medications that are usually not covered by JKN (line 385- 387)

7. Page 12: Despite being uncovered, what explains uninsured to spend less in urban than in rural areas?

Thank you for your feedback. 56% of Indonesians live in urban areas and the majority are in the middle-upper quintile, which means they have a strong preference to avoid generic medicines and prefer to pay OOP to receive quicker and better services. These preferences often exceed JKN benefits and, thus, this segment of the population is more likely to incur OOP. We have incorporated this into lines 308 – 312.

8. Page 13: What factors/reasons contribute to middle wealth quintile groups to obtain higher cost savings compared to poor and near-poor JKN population?

Thank you for this feedback, middle-income (Q3) households saved the most in OOP costs when compared to the uninsured (41% compared to Q1’s 38% and Q5’s 35%). This may be due to the middle class utilizing JKN more than the poor due to better knowledge of JKN benefits, and easier access to health care facilities contracted with BPJS-K. We have revised the narrative in line 383-387.

9. Page 13: If the benefit package and the cost associated with it are borne by JKN, what explains JKN households to experience more savings on OOP at public PHC facilities than private PHC facilities, especially for inpatient care? Can PHC handle the bulk of inpatient care? Table 1 indicated that only 1% of population accessed PHC inpatient services? If that is so, how important is this result?

More public PHC facilities are contracted with BPJS-K and thus the cost saving is higher. Also, more public PHC facilities provide inpatient care compared to private PHC ones. Although only 1% access inpatient care at public PHC, we believe that this result is important because it signals that the Indonesian social health insurance scheme that’s relatively new -JKN- is functioning in the right direction. The challenges that still persists in Indonesia is that many do not access health facilities even though they are sick. We added this information on line 252-254.

10. In general, overall reduction in OOP in Indonesia from 46% in 2012 to 32% (as % to THE) in 2019 may be the result of JKN and a large expansion of private health insurance as well. This needs to be observed and reported.

Thank you for your comment. This downward trend of OOP is most likely the result of JKN implementation, while private insurance scheme has been relatively constant in the last 10 years (15% of THE). We have revised the paragraph in line 460 – 462 to include your suggestion.

11. Page 7: Statistical Analysis: First line: This line appears to provide a wrong message. Rather “JKN ownership on OOP health spending” should have been “JKN enrolment on OOP health spending”. The literature in this area specifies insurance enrolees rather than as owners of insurance.

Thank you for your advice, we strongly agree and have replaced the terminology on line 186, 226, and 450 .

12. Page 9: Is East Timor part of Indonesia or West Timor? Please check the second paragraph.

Thank you for your thorough observation. We meant Nusa Tenggara Island, which is different from West Timor. We have made the change on line 238.
