# Supplementary material for: How Jaminan Kesehatan Nasional (JKN) coverage influences out-of-pocket (OOP) payments by vulnerable populations in Indonesia
Source: PLOS Glob Public Health. 2022 Jul 7;2(7):e0000203. doi: 10.1371/journal.pgph.0000203 (PMC10021284; doi:10.1371/journal.pgph.0000203)
Supplement: Attachment — Submitted filename: Reviewers response_30May22.docx [file pgph.0000203.s005.docx]

Thank you to Reviewer #2 (Sakthivel Selvara) for your inputs in improving the quality of this paper.

Thank you also to Reviewer #1 (Indranil Mukhopadhyay) for your thorough read and astute comments and requests for clarification. We feel the comments and questions have helped us to clarify and, in some cases, simplify our descriptions. Please find our responses in blue to the feedback below.

Reviewer 1: The authors have not responded to all previous comments satisfactorily. For instance, using 'OOP expenses' without trying to adjust it by the ability to pay of the household (income/household consumption expenditure) remains a key limitation of the study. Despite pointing it out, the authors have not addressed this.

Thank you for your feedback. We took into account the expenditure variable when generating wealth quintiles. The steps are as follows:

First, annual households’ expenditure is transformed to real terms to ensure comparability across regions using districts’/city’s 2018 poverty line. We followed this transformation method from Ajay T, et al (2016). Second, we sorted the transformed expenditure variable from lowest to largest and divided them into 5 quintiles. Lastly, we included this wealth quintile variable as one of the key independent variables, which is interacted with the JKN/insurance enrolment variable. This, in our opinion, is sufficient to address the issue of ability to pay, as it is also supported by literatures in this area.

In addition, if the nominal expenditure is directly added as an independent variable in the model, there may be reversed causality with the dependent (OOP) due to strong correlation, and vice versa, which could weaken the analysis. Similarly, if we generate a variable comprising of the share of OOP against expenditure as the dependent, then there will be an issue with interpreting the results, especially with regards to policy. As we are actively engaged with policy makers and this study serves as the first attempt to understand household’s out-of-pocket in Indonesia and provide evidence for policy making.

Though the authors have explained the two-part model they have used in the methods section, they have not provided the regression results. In the absence of regression results it is not possible to verify the statements or observations made. the current paper essentially demonstrates the cross tabs and related figures, which are not adequate to support the results and subsequent discussions, particularly related to establishing any association!

Thank you for your feedback. In the supporting information file, we have provided the regression result (S3 Table) which numbers we referred to in the main analysis and discussions. This is the marginal result of the regression output (S2 Table) by using the *margin* command in Stata.

The authors have not explained the logic of using two rounds of SUSENAS survey and the methods applied to use the two rounds. Remains a major limitation of the study.

Thank you for your feedback. The two rounds were used to look at data consistencies as the 2018 Susenas is the first to include households’ OOP data. By comparing with the 2019 data, we made sure that data structures and quality are consistent. We found that the two years are consistent and are qualified to conduct a cross-sectional analysis. Furthermore, by using the two years, we could have higher numbers of observations which would be favourable for a more robust result, as it reaches nearer to the normal distribution, and could eliminate heteroskedasticity. We have added the additional information on line 150-154.

There are contradictions in the results presented and policy recommendations made. For instance, in line 476 the authors argue that Private hospitals should focus on curative services, while reporting repeatedly that OOP is much higher in private facilities!

Thank you for your feedback. You raised a good point. In this version, we have tried to further explain the roles of public PHC, private PHC, as well as private hospitals in reducing OOP (line 500-513).

- Public PHC facilities are currently focusing on curative services, and less on promotional and preventive efforts due to the majority of JKN members being registered at these facilities. Approximately 83.2% of JKN members are registered at public PHC facilities while only 16.8% were registered at private ones in 2020. Therefore, workloads at public PHCs should be optimally balanced through redistributing some of their JKN members to private PHC. Consequently, there will be stronger promotive and preventive services at public PHC facilities that attract, and screen patients early could prevent higher admission to hospital, hence will have tremendous potential to reduce OOP spending.
- JKN’s rates should be adjusted to further incentivize private PHC facilities to network with BPJS-K. By 2021, there were only 67% networked private PHC, in other words redistributing members may be challenging in some areas with no or little contracted private PHC.
- Efforts to reduce OOP should be followed by contracting more private hospitals as it only accounts for 83% of total private hospital nationwide in 2020. This is important because the number of public hospitals is only 931, nearly half of the private. In order to attract more private hospital, rates should be adjusted to current prices as they have not been updated in the past 6 years.

Some specific issues:
Line 174: the variable "at least one household member feeling sick in the past month" "can be a proxy controlling for chronic illness"! This is surprising! needs better explanation!

Thank you for your feedback. We may have picked an inaccurate term. What we meant is that this variable can capture households with relatively high OOP health spending due to illness. Ideally, there is a control variable that signals chronic illness, but this is not available in Susenas. We revised the sentence in line 193 – 195.

line 298: The unconditional effect should take into account households who have any event of illness, rather than all households.

Thank you for your feedback. From our understanding of your feedback, we already did control for illness in the regression model (line 193-195). In regard to the unconditional effect, as explained by Buntin MB, Zaslavsky AM (2004) and also Matsaganis M, Mitrakos T, Tsakloglou (2009), to obtain the unconditional predicted OOP estimate (i.e. how much a household spends OOP when obtaining healthcare), the probabilities of paying OOP from the first part are multiplied by the expected levels of OOP from the second part. This refers to the population level estimate (all households). In addition, as JKN is a social insurance and covers preventive and promotive services -not only curative- the effect of it may also be seen among households with no illnesses (line 226-228).

As Susenas 2018 is the first study in Indonesia to provide OOP data, our goal was to first assess whether JKN is on the right track since its implementation in 2014 by estimating its influence on health services that are in the benefit package based on wealth quintiles, health providers, and regions. We believe that this finding would encourage improvements in the JKN system to achieve UHC and gives financial protection. Looking at specific households with any event of illness will be a potential topic for our next study. Merging Susenas with other datasets containing frequency and severity of illness will be required to make a deeper analysis. But we think it goes beyond the narrower scope of this study.

line 310: "middle-upper quintile, and they seem to have a strong preference to avoid generic medicines..." such statements, if not drawn from the study findings, need to be backed up by references.

Thank you for the reminder, we missed the reference and have added it in line 337.

Line 313: Urban areas have lower OOP because of more facilities...: such observations are contradictory to the vast literature on induced demand which shows that more facilities and greater ability to pay increases prices in urban areas. need references.

Thank you for this feedback. The uninsured households experience 12% less OOP payments in urban areas versus rural ones. There are 2 mechanisms that take place in urban and rural, that we are explaining in this version (line 340-351).

- In rural areas, delays in access to healthcare are persistent, causing patients to incur higher cost of treatment (Mahmood, Mohammad Afzal, et al, 2021). This delay may be caused by less availability of facilities and based on our data analysis (using Susenas 2018 and 2019), almost half of the uninsured living in rural areas are the poor (quintile 1 and 2). This is again confirmed by our analysis that the proportion of uninsured households in rural areas that did not access healthcare facilities even when they are sick are 9% higher than that who live in urban areas.
- On the other hand, in urban areas with higher provider density, the magnitude of supply-induced demand is weaker among poor patients (Bir and Eggleston, 2003). For example, doctor’s dual practices in urban areas will only concentrate inducement effort by self-referring to their more expensive facility, on those most likely to respond, which are those with higher ability to pay. However, in our case, the uninsured poor in urban areas account for 45.5% (quintile 1 and 2). Therefore, OOP in rural is higher than in urban among the uninsured.
